# Antiretroviral therapy resistance mutations among HIV infected people in Kazakhstan

**DOI:** 10.1038/s41598-022-22163-7

**Published:** 2022-10-13

**Authors:** Ainur Mukhatayeva, Aidana Mustafa, Natalya Dzissyuk, Alpamys Issanov, Zhussipbek Mukhatayev, Bauyrzhan Bayserkin, Sten H. Vermund, Syed Ali

**Affiliations:** 1 Department of Biomedical Sciences, Nazarbayev School of Medicine, Nazarbayev University, Astana, Kazakhstan; 2 Kazakh Scientific Center of Dermatology and Infectious Diseases, Almaty, Kazakhstan; 3 School of Population and Public Health, University of British Columbia, Vancouver, Canada; 4 Yale School of Public Health, New Haven, CT USA

**Keywords:** Microbiology, Risk factors, Signs and symptoms

## Abstract

In Kazakhstan, the number of people living with HIV (PLHIV) has increased steadily by 39% since 2010. Development of antiretroviral therapy (ART) resistance mutations (ARTRM) is a major hurdle in achieving effective treatment and prevention against HIV. Using HIV *pol* sequences from 602 PLHIV from Kazakhstan, we analyzed ARTRMs for their association with factors that may promote development of ARTRMs. 56% PLHIV were infected with HIV subtype A6 and 42% with CRF02_AG. The ARTRM Q174K was associated with increased viral load and decreased CD4+ cell count, while infection with CRF02_AG was associated with a lower likelihood of Q174K. Interestingly, CRF02_AG was positively associated with the ARTRM L10V that, in turn, was observed frequently with darunavir administration. Infection with CRF02_AG was positively associated with the ARTRM S162A that, in turn, was frequently observed with the administration of nevirapine, also associated with lower CD4 counts. Zidovudine or Nevirapine receipt was associated with the development of the ARTRM E138A, that, in turn, was associated with lower CD4 counts. Determination of a patient’s HIV variant can help guide ART choice in Kazakhstan. For example, PLHIV infected with CRF02_AG will benefit less from darunavir and nevirapine, and emtricitabine should replace zidovudine.

## Introduction

The estimated number of newly diagnosed persons living with HIV (PLHIV) declined from 2.8 million in 2000 to 1.5 million in 2020^1^. However, Eastern Europe and Central Asia are exceptions and between 2010 and 2020, an estimated 43% increase in new HIV infections was reported^2^. In Kazakhstan, the number of PLHIV was 22,000 in 2016, increasing to an estimated 24,000 in 2017, 26,000 in 2018, 33,000 in 2019, and 35,000 in 2020. Kazakhstan’s newly reported HIV cases grew 73% between 2010 and 2020^3^.

The antiretroviral (ARV) treatment is recommended by the U.S. Department of Health and Human Services (DHHS) and the World Health Organization (WHO) for treatment of all PLHIV^4^. In accordance with WHO recommendations, the Ministry of Health in Kazakhstan has authorized a clinical protocol for HIV treatment to begin universal ART administered no later than 14 days from the date of diagnosis, regardless of the clinical stage of the disease or CD4+  cell count^5^. The first line regimen in Kazakhstan offers both a preferred ART combination or an alternative^6^. If one of the components of the preferable treatment is not suitable for PLHIV for any variety of reasons, then it can be changed to another—the alternative treatment. For instance, patients with exceptional circumstances, such as pregnant women, patients with low (≤ 50 cells/μL) CD4+ cells counts, PLHIV with co-morbid conditions like neurocognitive disorders, chronic kidney disease, cardiovascular diseases, or chronic hepatitis, may be prescribed tailored alternative ART regimens. Since 2017, the list of ARV drugs in Kazakhstan has been expanded: tenofovir (TDF), atazanavir (ATV) with ritonavir or cobicistat, bictegravir (BIC), elvitegravir, as well as these combination drugs: TDF/lamivudine (3TC)/ dolutegravir (DTG), TDF alafenamide/emtricitabine (FTC)/bictegravir, TDF/emtricitabine/elvitegravir/cobicistat, DTG/rilpivirine, and DTG/3TC. Preferred ART regimens for adults were changed from TDF/FTC/EFV to TDF/FTC + DTG or TDF/FTC/BIC, and for pregnant women was changed from TDF + 3TC (or FTC) + EFV to TDF / FTC + DTG. Similarly, the post-exposure prophylaxis regimen (TDF + 3TC (or FTC) + LPV/r was replaced by TDF (or TAF) + 3TC (or FTC) + DTG). Additionally, the treatment effectiveness indicators have been revised recently; in adults (instead of VL < 1000) and in children (instead of VL < 500) a new treatment efficacy indicator VL < 50 has been introduced that will allow timely testing of resistance and, based on that, a change in treatment regimen. Changes in the perinatal diagnostic algorithm now require the first biomaterial sampling to be done within 48 h after birth to accelerate diagnosis and initiation of early ART in the newborn^7^.

The Joint United Nations Program on HIV/AIDS (UNAIDS) has established a 90–90–90 strategy that sought to reduce the number of new HIV infections to 500,000 per year by 2020, with even more ambitious 95–95–95 goals for 2030^8^. To achieve the 2020 goal, 90% of all people living with HIV (PLHIV) would need to know their HIV status, 90% of all people with diagnosed HIV will have received sustained ART, and 90% of all people receiving ART will have been virally suppressed, i.e., viral load < 1000 copies/mL^9^. According to UNAIDS reports, as of 2020, in Kazakhstan, the efforts towards the 90–90–90 target have slowly increased to an estimated 78–73–84^10^.

To achieve UNAIDS 95–95–95 targets by 2030, it is imperative to control the development of ART resistance by maintaining adherence to treatment among PLHIV receiving ART. During replication, HIV produces a high rate of mutational variants that pose challenges to achieving effective ART therapy and viral suppression^11–14^. Factors that give rise to ART resistance mutations (ARTRM) include, sub-optimal treatment adherence, duration of viral replication maintained during incompletely suppressive therapy, effect of ARTRM on drug susceptibility and virus replication, and the ease of acquisition of a particular ARTRM^8^. ART resistance can be caused by the presence of acquired drug resistant (ADR) mutations in treatment-experienced PLHIV and/or transmitted drug resistance (TDR) mutations in treatment-naïve PLHIV^15,16^. Among populations receiving non-nucleoside reverse transcriptase inhibitors (NNRTI)-based ART, the reported levels of drug resistance can vary widely, NNRTIs from 50 to 97% and nucleoside reverse transcriptase inhibitors (NRTIs) from 21 to 91% in one review^17^.

In this study, we present the analysis of HIV drug resistance mutations among 602 PLHIV in Kazakhstan. We analyze associations between ARTRMs and contributing factors, such as CD4 count, viral load, ART, and infecting HIV subtype.

## Methods

Ethical approval for this study was obtained from Institutional Research Ethics Committee, Nazarbayev University, Kazakhstan (# 141/06052019). All experiments were performed in accordance with relevant guidelines and regulations. All participants gave informed consent to participate in the parent study from which *HIV pol* sequences from 602 PLHIV receiving ART were analyzed. All the PLHIV were registered with Kazakh Scientific Center of Dermatology and Infectious Diseases, Almaty, Kazakhstan. All patient information, including demographic data, was collected at the time of registration. Blood samples from the participants were collected between 2017 and 2019. Analyses for CD4+ cell counts were performed using BD FACS Count Reagent Kit (BD Biosciences, San Jose, USA) and for viral load using AmpliSens HIV-Monitor-FRT kit (Amplisens, Moscow, Russia). The AmpliSens HIV Resist-Seq kit (Amplisens, Moscow, Russia) was used for preparation of samples for sequencing. The kit includes reagents for RNA extraction from blood samples, RT-PCR, and specifically amplifies the *Pro* (protease) and *RT* (reverse transcriptase) genes using random primers provided with the kit. Following amplification, the samples were sequenced on Applied Biosystems 3100 Genetic Analyzer (Applied Biosystems, USA) with a usage of BigDye Terminator v1.1 Cycle Sequencing Kit (Applied Biosystems, USA).

All sequences were subtyped using REGA-HIV-1 automated subtyping tool^18^. Recombinant forms were further analyzed using the RIP tool^19^. The FASTA format file with sequences was uploaded to the HIVdb Program provided by the Stanford HIV Database^20,21^. Level of resistance was assigned to the ARTRM as follows: "Susceptible" implied no evidence of decreased ARV susceptibility. "Potential low-level resistance" implied presence of mutations that suggested previous ARV exposure or of mutations that were associated with drug resistance only when they occurred in the presence of other mutations. "Low-level resistance" was used to describe mutations that confer lower ARV susceptibility in vitro, or mutations that led to poor virological response to ARV treatment in a patient. "Intermediate resistance" signified high probability of a drug's activity being decreased, whereas "high-level resistance" meant the expected level of resistance was close to that seen in viruses with the highest levels of in vitro drug resistance, or that clinical evidence existed showing that PLHIV infected with these viruses had little to no virological response to ARV treatment^20,21^.

Statistical analyses were performed using *STATA* version 15.0 statistical software^22^. Continuous variables were summarized in means and standard deviations (SD) when normal distributions were noted or medians and interquartile ranges when distributions were skewed. Wilcoxon rank-sum test was used to evaluate the associations of these continuous variables: CD4 count, viral load, and the presence of ARTRMs. We used simple logistic regression to assess the relationship between the administration of a specific ARV drug or drug combination or a specific HIV subtype with the presence of ARTRMs. The association between the administration of specific ART component and CD4 count or viral load was analyzed using the Wilcoxon rank-sum test. Simple and multiple logistic regression analysis was performed to determine the direction and strength of association between independent variables and the occurrence of virologic failure. Crude and adjusted odds ratios (OR) with 95% confidence interval (CI) were calculated from univariate and multivariable analysis, respectively. A p-value < 0.05 was considered statistically significant. Among all ARTRMs, those drug resistance mutations possessing a significant association with continuous or categorical variables were analyzed.

## Results

### Demographics

Study participants comprised almost equally of men and women, coming from all regions of Kazakhstan: including Akmola, Aktobe, Atyrau, East Kazakhstan, Karaganda, Kostanay, Kyzylorda, Mangystau, North Kazakhstan, Pavlodar, Turkistan, West Kazakhstan, Zhambyl regions; and 3 cities with State status, i.e., Almaty, Shymkent, and Nur-Sultan. Samples, collected in 2017 (38%), 2018 (43%) and 2019 (19%), were all from ARV-experienced PLWH (Table 1).

**Table 1:** Demographic information about study participants.

	2017	2018	2019	Overall
**Sex**				
Male	114 (50%)	134 (52%)	63 (54%)	311 (52%)
Female	115 (50%)	125 (48%)	53 (45%)	293 (49%)
**Age**				
0–15	5 (2%)	1 (0.4%)	8 (7%)	14 (2%)
16–40	91 (40%)	93 (36%)	23 (20%)	207 (34%)
41–60	85 (37%)	138 (53%)	66 (57%)	289 (48%)
≥ 61	48 (21%)	26 (10%)	19 (16%)	93 (15%)
**Regions**				
Akmola	11 (5%)	17 (7%)	8 (7%)	36 (5%)
Aktobe	5 (2%)	2 (0.7%)	1 (1%)	8 (7%)
Almaty	52 (23%)	72 (28%)	24 (21%)	148 (24%)
Atyrau	2 (1%)	1 (0.4%)	0 (0%)	3 (2.5%)
East Kazakhstan	1 (0.4%)	11 (4%)	10 (8%)	22 (4%)
Karaganda	61 (27%)	43 (17%)	16 (14%)	120 (20%)
Kostanay	1 (0.4%)	1 (0.4%)	20 (17%)	22 (4%)
Kyzylorda	13 (6%)	4 (1.5%)	1 (1%)	18 (3%)
Mangystau	4 (2%)	11 (4%)	0 (0%)	15 (2%)
North Kazakhstan	9 (4%)	3 (1%)	3 (2.5%)	15 (2%)
Nur-Sultan	6 (3%)	5 (2%)	4 (3%)	15 (2%)
Pavlodar	24 (10%)	37 (14%)	4 (3%)	31 (5%)
Turkistan	20 (9%)	26 (10%)	20 (17%)	66 (11%)
West Kazakhstan	6 (3%)	7 (3%)	3 (2.5%)	16 (3%)
Zhambyl	14 (6%)	18 (7%)	2 (2%)	34 (6%)
**Subtype**				
A6	124 (54%)	143 (55%)	73 (63%)	564 (56%)
CRF02_AG	102 (44%)	110 (43%)	41 (35%)	393 (39%)
Other	4 (2%)	6 (2%)	1 (1%)	47 (5%)
**Risk factors**				
PWID	92 (40%)	94 (36%)	141 (34%)	327 (54%)
Heterosexual contact	51 (22%)	61 (24%)	53 (13%)	165 (27%)
MSM	0 (0%)	0 (0%)	3 (1%)	3 (0.5%)
Sex worker	2 (1%)	0 (0%)	1 (0.3%)	3 (0.5%)
Not registered	82 (36%)	104 (40%)	113 (27%)	299 (50%)
Overall	229 (38%)	258 (43%)	116 (19%)	602 (100%)

### ART regimens among participants

The Ministry of Health in Kazakhstan has established a clinical protocol for HIV treatment with preferable or with alternative ART regimens. The preferable regimen is two NRTIs plus an integrase inhibitor (INI) while the most common alternative is two NRTIs plus an NNRTI. Among our 602 study participants, only 37.5% were taking a Ministry of Health ART regimen (Table 2): 192 (32%) on TDF/FTC/EFV, 28 (5%) on ABC/3TC/EFV, 3 (0.5%) on TDF/FTC/DRV, and just 2 (0.3%) on the preferable TDF/FTC/DTG regiment. Most popular among other ART regimens were ZDV/3TC/NVP (n = 119 or 20%) and ZDV/3TC/EFV (n = 93 or 15.4%; Table 2).

**Table 2:** Association between CD4 count (cells/mm3), Viral load (copies/ml) and the type of ARV combination.

ARV classification	ARV combination	Number of PLWH	CD4 count (cells/mm^3^)			Viral load (copies/ml)			
			Low	Medium	High	Undetectable	Low	Medium	High
Only NRTI	ZDV 3TC ABC	15 (2.5%)	4 (0.7%)	7 (1.2%)	4 (0.7%)	0 (0.00%)	9 (1.5%)	4 (0.7%)	2 (0.33%)
	ZDV 3TC TDF	5 (0.8%)	0 (0.00%)	4 (0.7%)	1 (0.2%)	0 (0.00%)	0 (0.00%)	4 (0.7%)	1 (0.2%)
	TDF FTC ABC	3 (0.5%)	0 (0.00%)	3 (0.5%)	0 (0.00%)	0 (0.00%)	0 (0.00%)	3 (0.5%)	0 (0.00%)
NRTI NRTI NNRTI	ABC 3TC EFV (alternative)	28 (5%)	5 (0.8%)	20 (3.3%)	3 (0.5%)	0 (0.00%)	7 (1.2%)	14 (2.3%)	7 (1.2%)
	ABC 3TC NVP	14 (2.3%)	4 (0.7%)	9 (1.5%)	1 (0.2%)	0 (0.00%)	6 (1%)	2 (0.33%)	6 (1%)
	ZDV 3TC EFV	93 (15.4%)	32 (5.3%)	47 (7.8%)	14 (2.3%)	4 (0.7%)	28 (4.6%)	31 (5%)	30 (5%)
	ZDV 3TC NVP	119 (20%)	44 (7.3%)	58 (9.6%)	17 (2.8%)	2 (0.33%)	41 (7%)	42 (7%)	34 (5.6%)
	D4T 3TC EFV	1 (0.2%)	0 (0.00%)	1 (0.17%)	0 (0.00%)	0 (0.00%)	1 (0.2%)	0 (0.00%)	0 (0.00%)
	TDF FTC ETR	1 (0.2%)	1 (0.2%)	0 (0.00%)	0 (0.00%)	0 (0.00%)	0 (0.00%)	1 (0.2%)	0 (0.00%)
	TDF FTC EFV (alternative)	193 (32%)	55 (9%)	92 (15.3%)	46 (7.6%)	12 (2%)	55 (9%)	69 (11.5%)	57 (9.5%)
	TDF FTC NVP	10 (1.7%)	3 (0.5%)	6 (1%)	1 (0.2%)	0 (0.00%)	1 (0.2%)	3 (0.5%)	6 (1%)
NRTI NRTI PI	ABC 3TC DRV	5 (0.8%)	2 (0.33%)	3 (0.5%)	0 (0.00%)	0 (0.00%)	1 (0.2%)	0 (0.00%)	4 (0.7%)
	ABC 3TC LPV/r	16 (3%)	3 (0.5%)	9 (1.5%)	4 (0.7%)	1 (0.2%)	4 (0.7%)	6 (1%)	5 (0.8%)
	ABC FTC LPV/r	2 (0.33%)	0 (0.00%)	2 (0.33%)	0 (0.00%)	0 (0.00%)	1 (0.2%)	1 (0.2%)	0 (0.00%)
	ZDV 3TC LPV/r	54 (9%)	9 (1.5%)	33 (5.5%)	12 (2%)	1 (0.2%)	15 (2.5%)	23 (4%)	15 (2.5%)
	D4T 3TC LPV/r	1 (0.2%)	0 (0.00%)	1 (0.17%)	0 (0.00%)	0 (0.00%)	1 (0.2%)	0 (0.00%)	0 (0.00%)
	D4T FTC LPV/r	1 (0.2%)	0 (0.00%)	1 (0.17%)	0 (0.00%)	0 (0.00%)	0 (0.00%)	1 (0.2%)	0 (0.00%)
	TDF 3TC LPV/r	1 (0.2%)	1 (0.2%)	0 (0.00%)	0 (0.00%)	0 (0.00%)	0 (0.00%)	0 (0.00%)	1 (0.2%)
	TDF FTC DRV (alternative)	3 (0.5%)	0 (0.00%)	3 (0.5%)	0 (0.00%)	1 (0.2%)	1 (0.2%)	1 (0.2%)	0 (0.00%)
	TDF FTC LPV/r	31 (5%)	8 (1.3%)	15 (2.5%)	8 (1.3%)	2 (0.33%)	10 (1.7%)	11 (2%)	8 (1.3%)
NRTI NRTI INI	ABC 3TC DTG	3 (0.5%)	2 (0.33%)	1 (0.17%)	0 (0.00%)	0 (0.00%)	0 (0.00%)	1 (0.2%)	2 (0.33%)
	TDF FTC DTG (preferable)	2 (0.33%)	1 (0.2%)	1 (0.2%)	0 (0.00%)	1 (0.2%)	0 (0.00%)	0 (0.00%)	1 (0.2%)
	Total	602 (100%)	175 (29%)	316 (52.5%)	111 (18.4%)	24 (4%)	181 (30%)	217 (36%)	179 (30%)

*PI* protease inhibitors, *NRTI* nucleoside reverse transcripts inhibitors, *NNRTI* non-nucleoside reverse transcripts inhibitors, *INI* integrase inhibitors.

### CD4+ cell count and viral load

CD4 count was classified into 3 categories: low < 200 cells/mm^3^, medium 200–500 cells/mm^3^, high > 500 cells/mm^3^. Viral load was categorized as follows: undetectable < 400 copies/ml, low 400–10,000 copies/ml, medium 10,001–100,000 copies/ml, high > 100,000 copies/ml (Table 2). We found low CD4 counts < 200 cells/mm^3^ in 175 (29%) of study participants at the time of enrolment. Medium CD4 counts of 200–500 cells/mm^3^ were seen in 316 (52.5%), and > 500 cells/mm^3^ in 111 (18%) participants (Table 2). Only 24 (4%) participants had undetectable viral load (below 400 copies/ml). We found < 1000 viral copies/ml in 181 (30%), 10,001–100,000 copies/ml in 217 (36%), and > 100,000 copies/ml in 179 (30%) participants (Table 2).

### Occurrence of drug resistance mutations

Based on analysis carried out using the Stanford HIV database, 191 of 602 (32%) sequences showed the presence of drug resistance mutations. The distribution of ART resistance was: 11 (2%) PI, 21 (3%) NRTI, 108 (18%) NNRTI, 1 (0.2%) PI and NRTI, 3 (0.4%) PI and NNRTI, 47 (8%) NRTI and NNRTI.

Drug resistance mutations were divergent in two viral subtypes: A6 and CRF02_AG (Supplementary Table 1). For instance, 132 (39%) patients infected with A6 subtype exhibited the A62V NRTI accessory mutation, whereas only 8 (3%) patients carrying CRF02_AG subtype had this mutation. In contrast, 240 (94%) CRF02_AG-infected patients exhibited S162A RT mutation, while only 2% of A6 patients had this mutation.

Both subtypes showed an equal distribution (23%) of M184V mutation and similar distributions of Q174K: 90% occurrence in A6, and 83% in CRF02_AG. As seen in an earlier study in Kazakhstan^23^, the V77I mutation was observed frequently (26%) in A6 subtype patients. Mutation K103N was found to less often (15%) among the A6 subtype compared to CRF02_AG (40%) (Supplementary Table 1). Other mutations were much less common in the two subtypes: A6: G190S (12%), K65R (4%), K101E (3%), E138A (5%), L10V (4%), E138A (5%); CRF02_AG: K65R (3%), E138A (3%), Y181C (2%), L10V (10%).

### Antiretroviral drug resistance

#### Protease inhibitors

**Figure 1:**
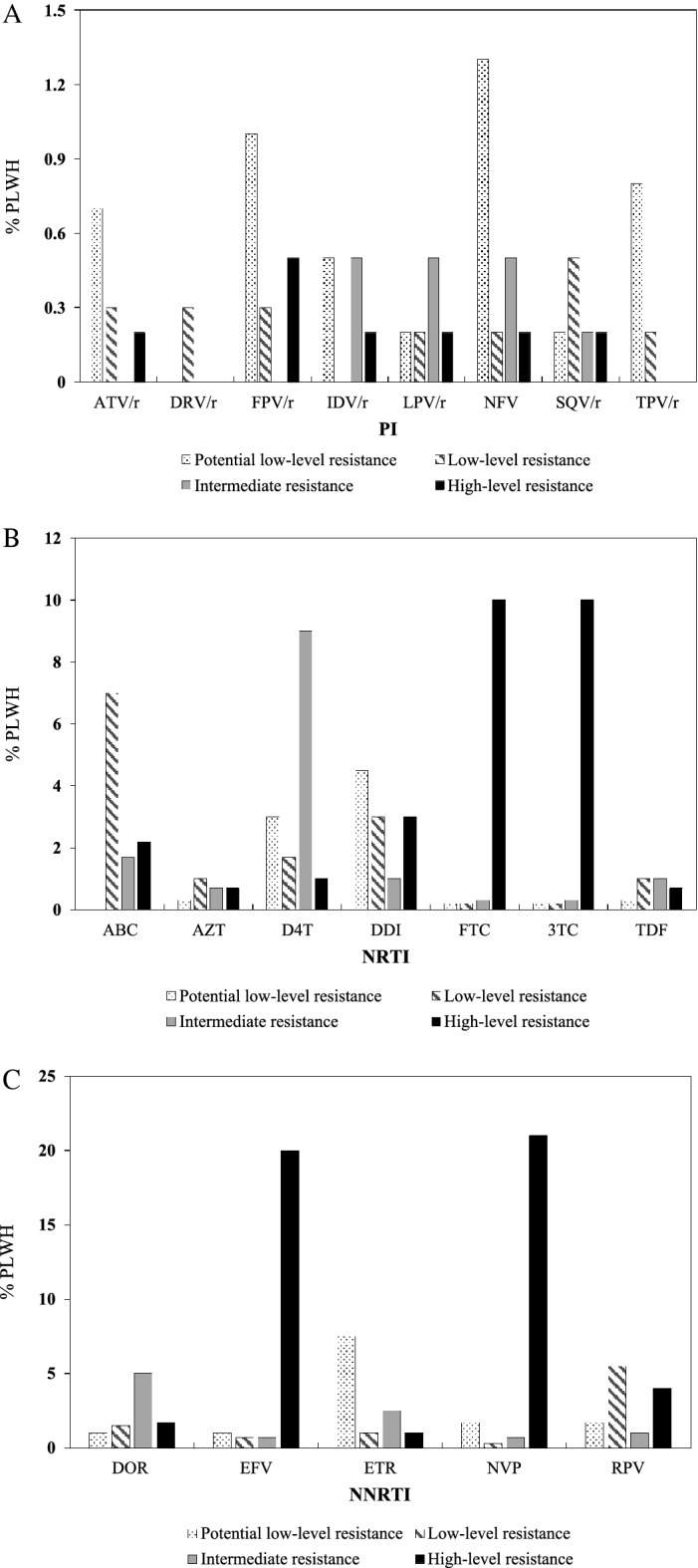
Antiretroviral drug resistance: (**A**) PI. *r* ritonavir-boosted, *ATV* atazanavir, *DRV* darunavir, *FPV* fosamprenavir, *IDV* indinavir, *LPV* lopinavir, *NFV* nelfinavir, *SQV* saquinavir, *TPV* tipranavir. (**B**) NRTI. *ABC* abacavir, *ZDV* zidovudine, *D4T* stavudine, *DDI* didanosine, *FTC* emtricitabine, *3TC* lamivudine, *TDF* tenofovir disoproxil fumarate. (**C**) NNRTI. *DOR* doravirine, *EFV* efavirenz, *ETR* etravirine, *NVP* nevirapine, *RPV* rilpivirine;

Among our study group, fewer PLHIV were found resistant to PI than those to NRTI or NNRTI (Fig. 1). There were three (0.5%) PLHIV with high level resistance to FPV/r, two (0.3%) for NFV, and one (0.2%) each for ATV/r, IDV/r, LPV/r, and SQV/r (Fig. 1A). Intermediate resistance for IDV/r, LPV/r, or NFV was found among three (0.5%), and for SQV/r in one (0.2%) patient. Low-level resistance was found for SQV/r among three, for FPV/r, DRV/r, and ATV/r among two (0.3%), and for LPV/r, NFV, and TPV/r in one (0.2%) patient (Fig. 1).

#### NRTI

We found 59 (10%) PLHIV with high level resistance to FTC or 3TC. Other resistance was far less common: 15 (3%) to DDI, 13 (2.2%) to ABC, 6 (1%) to D4T, 4 (0.7%) to ZDV, and 4 (0.7%) to TDF (Fig. 1B). Other levels of resistance were observed among the participants as follows: Intermediate: D4T (9%), ABC (1.7%), DDI (1%), TDF (1%), ZDV (0.7%), FTC (0.3%), 3TC (0.3%); low-level: ABC (7%), DDI (3%), D4T (1.7%), ZDV (1%), TDF (1%), 3TC (0.2%), FTC (0.2%), potential low-level: DDI (4.5%), D4T (3%), ZDV (0.3%), TDF (0.3%), 3TC (0.2%), FTC (0.2%) (Fig. 1B).

#### NNRTI

Among the study participants, different levels of resistance to NNRTI were observed as follows: High level: NVP (21%), EFV (20%), RPV (4%), DOR (1.7%), ETR (1%) (Fig. 1C); intermediate: DOR (5%), ETR (2.5%), RPV (1%), NVP (0.7%), EFV (0/7%); low-level: RPV (5.5%), DOR (1.5%), ETR (1%), EFV (0.7%), NVP (0.3%); potential low-level: ETR (7.5%), NVP (1.7%), RPV (1.7%), EFV (1%), DOR (1%) (Fig. 1C).

### Association of ARTRMs with CD4 count, viral load, ART components and HIV subtypes

**Figure 2:**
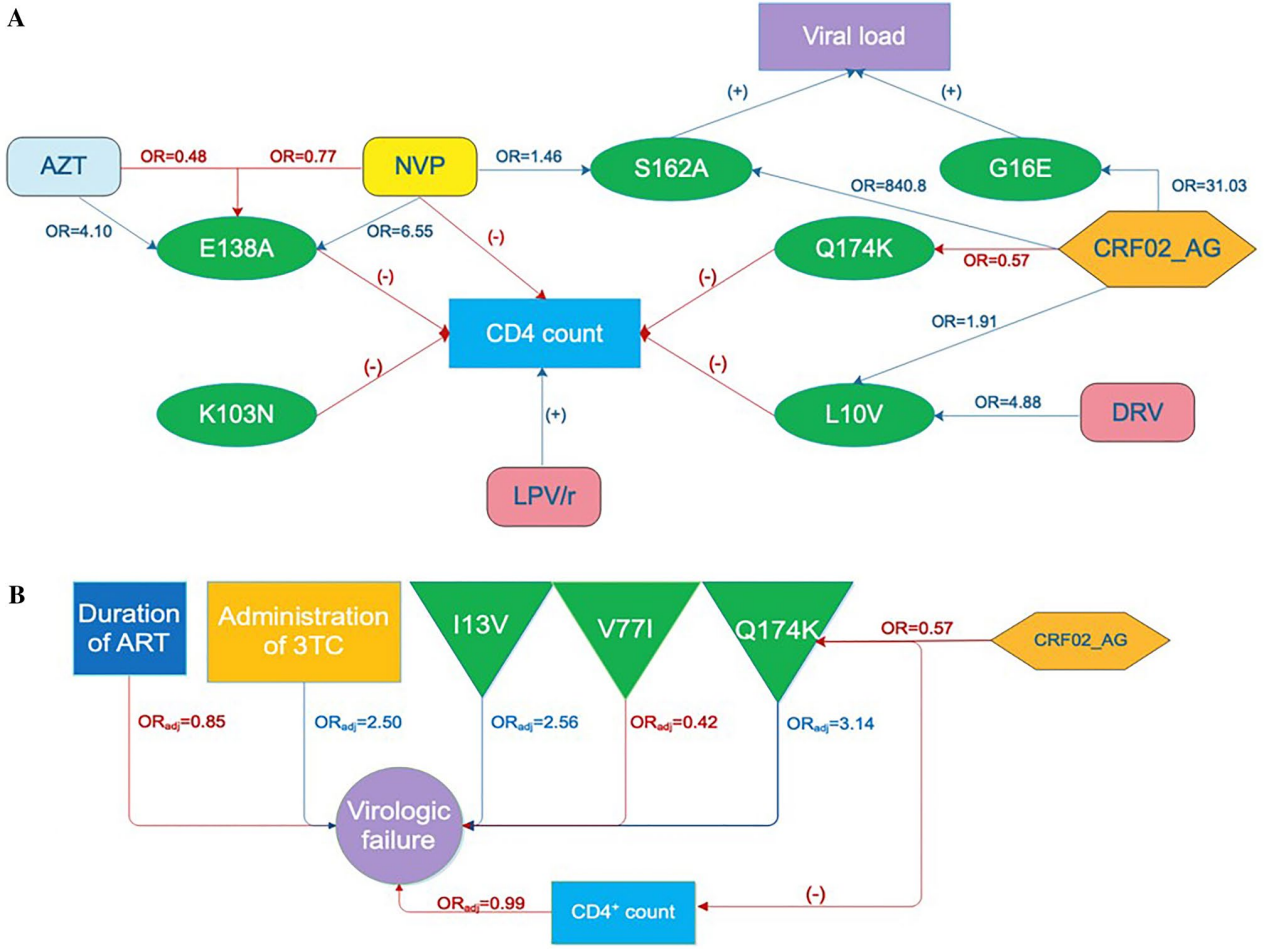
(**A**) Summary of the statistically significant associations between CD4 count, Viral load, ART components, Subtype and single mutations. Zidovudine (ZDV) is NRTI (light blue); Neviarpine (NVP) is NNRTI (yellow); Darunavir (DRV) and Lopinavir/Ritonavir (LPV/r) are PIs (pink); CRF02_AG is a subtype (orange); E138A, K103N, Q174K, S162A are RT ARTRMs (green), L10V, G16E are PI ARTRMs (green); OR odds ratio; (+) positive and (−) negative association from t-test (blue and red arrow, respectively. (**B**) A schematic representation of the effect of factors on the presence of virologic failure. I13V, V77I, Q174K are ARTRMs (green); OR_adj_ adjusted odds ratio from multivariable analysis.

Based on our results, only the ARTRMs showing significant associations with individual continuous or categorical variables (Supplementary Tables 2 and 3) were analyzed. While no statistically significant association was found between the duration of ART and sex, we found a statistically significant decrease in CD4 count among PLHIV with RT ARTRMs: E138A (accessory), K103N (major), and Q174K (polymorphic other), and protease ARTRM: L10V (accessory polymorphic), compared to those without these ARTRMs (p-value < 0.05) (Supplementary Table 2 and Fig. 2A). Furthermore, there was a significantly higher viral load among PLHIV with Q174K mutation in comparison to the PLHIV without this ARTRM.

When ARTRMs were analyzed in relation to ART components, we found that the odds of having E138A mutation was 4.10 and 6.55 times higher among the PLHIV who were taking Zidovudine (ZDV) and Nevirapine (NVP), respectively (OR 4.10; CI 1.24–13.53 and OR 6.55; CI 1.14–37.74). On the other hand, we found an interaction between the simultaneous administration of ZDV with NVP and the presence of E138A mutation, which would decrease the odds of possessing this ARTRM (OR with NVP among PLHIV taking ZDV 0.48; CI 0.09–2.65 and OR with ZDV in PLHIV taking NVP 0.77; CI 0.25–2.36). Moreover, the prescription of Darunavir (DRV), a protease inhibitor, was associated with a 4.9-fold increase in the odds of acquiring L10V mutation (OR 4.88; CI 1.09–21.66). The occurrence of RT ARTRM, S162A (other), was 1.46 times higher among PLHIV receiving NVP (OR 1.46; CI 1.002–2.13). Comparing various subtypes of the HIV-1 virus with the presence of ARTRMs, infection with CRF02_AG was found to increase the likelihood of L10V, G16E (protease, other) and S162A ARTRMs (OR 1.91; CI 1.02–3.55, OR 31.03; CI 18.60–51.76 and OR 840.79; CI 328.5–2152), while reducing the rate of Q174K mutation (OR 0.57; CI 0.36–0.93). There was a negative association with the administration of NVP and CD4 count, while the prescription with Lopinavir/ritonavir (LPV/r) had a positive association with CD4 count (Supplementary Table 3 and Fig. 2A).

### Analysis of factors associated with virologic failure

We defined virologic failure was defined as viral copies > 1000 copies/ml at a single visit. For the majority of study participants, data on follow-up measurement and adherence, as per the 2016 WHO consolidated antiretroviral guidelines^24^, were not available. Our univariate logistic analysis showed that, as expected, every 1 cell/mm^3^ increase in CD4 count was associated with a 1% decrease in the occurrence of virologic failure (OR 0.99; CI 0.997–0.999). In contrast, the presence of I13V, N37D, Q174K mutations increased, while I93L and V77I mutations reduced the odds of having virologic failure among the PLHIV (Supplementary Table 4 and Fig. 2B). In the multivariable model, an increase in the duration of ART was negatively associated with the development of virologic failure (OR 0.85; CI 0.76–0.95). Furthermore, the administration of 3TC increased the likelihood of virologic failure by 2.5 times (OR 2.50; CI 1.32–4.71). The presence of ARTRMs, I13V and Q174K, was associated with a 2.6 and 3.1-fold increase, respectively, in a viral load > 1000 copies/mL, while PLHIV who had V77I mutation had an approximately 60% decrease in the occurrence of virologic failure (OR 0.42; CI 0.21–0.86) (Supplementary Table 4 and Fig. 2B).

## Discussion

In 602 PLHIV receiving ART from all regions of Kazakhstan, drug resistance mutations were common. We have examined complex associations of specific ARTRMs with CD4 counts, viral loads, ART components, and HIV subtypes.

### Subtypes

Among our study participants 56% were infected with HIV subtype A6, 24% with CRF02_AG, 1.5% with subtype G, and 0.7% with other variants. Subtype A6 evolved as an indigenous HIV variant in former Soviet Union countries and is still predominantly prevalent in this region, including Kazakhstan^25,26^. It is now known that certain ARTRMs are more prone to be found in particular subtypes. For instance, K64R, a major mutation that induces high-level resistance to most NRTI/NtRTIs, is found to be particular for HIV-1 subtype C^27^. Likewise, some studies revealed that certain subtypes (subtype B) may have associations with specific ARV therapy response^28^. No such reports are available about the prevalence of ARTRM, or their association with the HIV variants prevalent, in Kazakhstan. A 2007 publication reported that 59% of HIV subtype A sequences derived from Russia and Kazakhstan PLHIV carried a secondary mutation, V77I, in protease^23^. Consistent with this earlier observation, 93 (15%) of our study participants carried the V77I mutation in the protease gene, with 89 (96%) of them infected with subtype A6 subtype.

### ARV distribution

In Kazakhstan, preferable or alternative ARV combination are prescribed for HIV positive patients^6^. Among out study participants, 32% were receiving the alternative combination, TDF FTC EFV regimen, while 20%, 15%, and 1.5% received other regimens, respectively, ZDV 3TC NVP, ZDV 3TC EFV, and TDF FTC NVP, which were neither preferable, nor alternative (Table 2). D4T and ZDV are not recommended as first-line therapy in high income nations. However, in most countries of Asia, the most prescribed first-line regimens still contain either D4T or ZDV, since they are more affordable^29–31^.

All currently available ARV drugs involve risk of ARTRM development^17^. For the frequently prescribed alternative first-line regimen based on EFV in combination with two NRTIs, usually TDF and either 3TC or FTC^24^, ARTRM have been reported more frequently for combinations containing EFV^17,24,32–36^.The main drawback of EFV is the predisposition to the single amino acid mutations, such as K103N, V106M, leading to high level of drug resistance^38–40^. ARTRM formation leads to a decrease in the virological response^41^, enhancing the risk of transmission of mutated variant to the newly infected^42,43^, increasing the chances of multidrug resistance in drug-naïve PLHIV^44^. It is, therefore, highly recommended to perform drug resistance testing before starting the ARV therapy^45,46^.

### The occurrence of drug resistance mutations

Among our study participants, ARV resistance mutations were seen in persons on PI + NRTI (0.2%), PI + NNRTI 0.4%), PI (2%), NRTI (3%), NRTI + NNRTI (8%), and NNRTI (18%). Recent studies report that rates of ARV resistance rise with time on ARV^47^. We found that virologic failure (viral copies > 1000 copies/ml as per 2016 WHO consolidated antiretroviral guidelines^24^), was found to be decreased by an increase in the duration of ART, higher CD4 count, and the presence of a V77I mutation (Fig. 2B).

We found that in our study cohort, some mutations occurred especially often: NRTI accessory A62V (22%), NNRTI major K103N (17%), and NRTI major M184V (8%). M184V is an NRTI mutation known to occur frequently among ARV experienced individuals. It induces high level resistance to 3TC. K103N is another persistent NNRTI mutation that gives rise to high-level resistance to NVP and variable resistance to EFV^48^. The A62V mutation is known to frequently co-occur with K65R and Q151M, giving rise to NRTI multi-resistance^49,50^. A62V was previously reported to be prevalent in HIV subtype A patients from Russia^51^; we observed a high occurrence of the same mutation in our study participants infected with subtype A6. Overall, there were 133 PLHIV carrying A62V mutation, 126 (95%) of whom were infected with subtype A6.

### Drug resistance scores

We found levels of resistance to NRTI and/or NNRTI to be higher than resistance to PI (Fig. 1), likely explained by the fact that the majority of PLHIV were under the ARV treatment in such combination as two NRTIs + NNRTI (76%) (Table 2), whereas 19% of PLHIV were taking two NRTIs + PI, only NRTIs (3%), and two NRTIs + INI (0.8%). A major reason for the development of ARV resistance mutations is insufficient adherence to the ARV regimen. Typically associated with difficulties in accessing healthcare, stigmatization of HIV, co-morbidities like drug addiction, and a shortage of medications due to prohibitive cost or other factors^52^.

### Associations between ARV mutations and factors that promote them

We found a significant association of presence of ARV mutations E138A, K103N, Q174K and L10V, with a decrease in CD4 count (Supplementary Table 2). Furthermore, the odds of E138A, a polymorphic mutation in the viral RT region, were found to be increased with ZDV or NVP use^20^. Surprisingly, the simultaneous administration of these drugs was negatively associated with the presence of E138A (Supplementary Table 2), suggesting that the administration of ART combinations containing both ZDV and NVP might be potentially beneficial in decreasing the odds of E138A emergence.

Another mutation in the PI gene, L10V, a polymorphic accessory PI mutation, was found to be favored by the prescription of DRV, a protease inhibitor. The same mutation has been reported to reduce PI susceptibility or contribute to the viral replication in collaboration with other PI-resistance ARTRMs^53,54^. One intriguing finding was that our analysis showed that CRF02_AG infection was associated with an increased likelihood of L10V and G16E mutations. A study by Udeze et al. also showed a higher frequency of these ARTRMs among PLHIV infected with CRF02_AG compared to other HIV variants^55^. Similarly, the risk of the occurrence of RT mutation, S162A, was found to be increased by the administration of NVP and with CRF02_AG infection.

For the majority of study participants, data on follow-up measurements were not available. Basing ART decisions on single viral load measurements may allow to establish treatment failure earlier—avoiding delay in switching ART regimen, and preventing the possible complications due to prolonged virologic failure. Indeed, there is evidence that switching the ART regimen following a single viral load > 1000 copies/mL could prevent approximately 10,215 deaths annually in South Africa, which supports the recent WHO guidelines recommending to use single viral load measurements for ART switch among patients receiving NNRTI-based regimen^56,57^

In our analysis, virologic failure was found to be decreased by an increase in the duration of ART, the number of CD4 cells and the presence of V77I mutation (Fig. 2B). The possible explanation for this can be an early occurrence of drug resistance and virologic failure, closer to the initiation of therapy. Furthermore, in another study V77I has also been associated with increased virologic response among PLHIV receiving fosamprenavir/ritonavir^58^.

In contrast, we found that the administration of 3TC and the presence of ARTRMs I13V and Q174K were found to increase the odds of virologic failure. Previous studies have also reported a lower occurrence of resistance mutations with FTC-containing ART combinations compared to those containing 3TC^59–62^. A possible explanation presented for this was the higher potency or longer duration of action of FTC compared to 3TC. Furthermore, the Dutch nationwide ATHENA cohort study concluded that 3TC increases the probability of virologic failure, whereas the use of FTC instead of 3TC was associated with a better virologic response^63^. The same study concluded that due to lack of sufficient evidence, the outcomes of ATHENA cohort study were inconclusive. Hence, current guidelines recommend that 3TC and FTC are interchangeable in ART therapy^64^.

The primary limitation to the generalization of these results was our limited sample size where ARV resistance mutations were determined in a one-time collected sample. Further studies with an expanded number of study participants, with samples collected periodically over a longer period of time, will provide a clearer picture of the evolution of ARV resistance and transmission across Kazakhstani populations. In our study group, data on adherence and follow-up viral load measurement were not available. Furthermore, all the participants were ARV experienced, which made it impossible to analyze transmitted mutations. Further studies need to be designed to investigate ARV mutations among drug naïve patients.

As with all clinical epidemiology studies, we must consider threats to internal and external (generalization) validity of our results based on potential sources of bias: selection, measurements, confounding, and analysis. Since ART access is relatively centralized and the Kazakh Scientific Center of Dermatology and Infectious Diseases in Almaty provided samples from patients throughout the nation, we believe that geographic generalizability is substantial. However, the preponderance of our participants were persons who inject drugs or persons with likely heterosexually acquired HIV infection. Underrepresentation of men who have sex with men should be noted, and we cannot discount misclassification among men who may deny same sex contact when they might, in fact, have occurred. Our data from 2017 to 2019 may not reflect current trends. Fortunately, measurements were state-of-the-art and subject to best laboratory practices. Use of multivariable models in our statistical analysis enabled examination of confounding factors and interactions. However, we acknowledge that 602 subjects are not enough to completely disentangle the relationships of sociodemographic and behavioral factors and ARV drug combinations with the many ARV mutations studied. Still our study contributes substantially to the Central Asia data in this sphere.

In conclusion, to achieve effective treatment and prevent further transmission of HIV in Kazakhstan, it is imperative that current treatment protocols are optimized to eliminate components of ARV combination that may promote ARTRM. For efficient ART optimization, complete patient profiling, including the existence of ARTRM and the infecting HIV subtype or variant, needs to be taken into account.

## Supplementary Information

Supplementary Tables.

## Data Availability

All data generated or analyzed during this study are included in the published article [and supplementary files].
