# Supplementary material for: Antiretroviral therapy resistance mutations among HIV infected people in Kazakhstan
Source: Sci Rep. 2022 Oct 13;12:17195. doi: 10.1038/s41598-022-22163-7 (PMC9562405; doi:10.1038/s41598-022-22163-7)
Supplement: Supplementary file 1 — Supplementary Tables. [file 41598_2022_22163_MOESM1_ESM.docx]

**Supplementary Table 1.**

| ART mutation | Mutation classification | Number of PLHIV (frequency) |
| --- | --- | --- |
| **A6 subtype (n=337)** | | |
| A62V | NRTI accessory | 132 (39%) |
| M184V | NRTI major | 76 (23%) |
| G190S | NNRTI major | 42 (12%) |
| K65R | NRTI major | 16 (4%) |
| K101E | NNRTI accessory | 11 (3%) |
| E138A | NNRTI accessory | 17 (5%) |
| K103N | NNRTI major | 50 (15%) |
| Q174K | Polymorphic RT | 303 (90%) |
| L10V | Polymorphic protease | 14 (4%) |
| S162A | RT mutation | 7 (2%) |
| E138A | NNRTI accessory | 17 (5%) |
| V77I | Protease mutation | 89 (26%) |
| **CRF02_AG subtype (n=254)** | | |
| A62V | NRTI accessory | 8 (3%) |
| K65R | NRTI major | 8 (3%) |
| M184V | NRTI major | 59 (23%) |
| E138A | NNRTI accessory | 8 (3%) |
| K103N | NNRTI major | 103 (40%) |
| Y181C | NNRTI major | 5 (2%) |
| Q174K | Polymorphic RT | 211 (83%) |
| L10V | Polymorphic protease | 25 (10%) |
| S162A | RT mutation | 240 (94%) |

**Supplementary Table 2.**

| **Categories** | **E138A** | | **K103N** | | **Q174K** | | **L10V** | | **G16E** | | **S162A** | |
| --- | --- | --- | --- | --- | --- | --- | --- | --- | --- | --- | --- | --- |
| **CD4 count**, cells/mm^3^ | (+) | (-) | (+) | (-) | (+) | (-) | (+) | (-) | (+) | (-) | (+) | (-) |
| Mean±SD | 227.8±186.5 | 336.1±228.1 | 248.4±156.6 | 343.2±32.8 | 324.5±232.5 | 383.5±186.0 | 282.2±240.5 | 336.5±226.2 | 328.1±229.4 | 337.9±225.4 | 319.1±231.8 | 342.4±224.2 |
| Median (IQR) | 187.5 (101-325) | 295.5 (181-449) | 217.5 (136-303) | 305.5 (185-452) | 280 (164-433) | 365 (243-529) | 230 (115-340) | 298 (183-450) | 291 (161-438) | 299.5 (196.5-450.5) | 267.5 (144-439) | 303.5 (205-449) |
| p-value | **0.02** | | **<0.01** | | **<0.01** | | **0.04** | | 0.43 | | 0.07 | |
| **Viral load,** ×10^3^ copies/mL | (+) | (-) | (+) | (-) | (+) | (-) | (+) | (-) | (+) | (-) | (+) | (-) |
| Mean±SD | 136.5±328.7 | 261.6±1048.6 | 224.3±598.5 | 261.7±1075.9 | 276.4±1092.8 | 135.8±484.6 | 410.9±1372.6 | 245± 1000.9 | 278.1±1273.5 | 231.7±614.5 | 262.9±1269.1 | 253.4±817.3 |
| Median (IQR) | 12.5 (3.85-65.0) | 28 (5.4-150) | 31.8 (9.3-158) | 26.2 (5.2-144) | 28.5 (5.9-156) | 16.1 (2.4-60.6) | 47 (9.3-170) | 26 (5.3-148) | 22.3 (4.3-124) | 34.3 (8.3-173) | 19.5 (4.2-107.7) | 33.5 (7.7-170) |
| p-value | 0.28 | | 0.51 | | **0.01** | | 0.38 | | **0.02** | | **0.01** | |
| **ART component administered^a^, OR (95% CI)** | | | | | | | | | | | | |
| NRTI | | | | | | | | | | | | |
| **ABC** | 1.65 (0.57-4.81) | | 0.59 (0.25-1.36) | | 1.17 (0.59-2.34) | | 0.98 (0.41-2.31) | | 1.07 (0.68-1.69) | | 1.03 (0.65-1.62) | |
| **AZT** | **Without NVP: OR 4.10 (CI 1.24-13.5)**  **With NVP: OR 0.48 (CI 0.09-2.65)*** | | 0.92 (0.56-1.52) | | 1.03 (0.64-1.64) | | 0.97 (0.53-1.77) | | 1.12 (0.81-1.55) | | 1.09 (0.79-1.51) | |
| **3TC** | 2.01 (0.75-5.38) | | 0.71 (0.43-1.17) | | 0.99 (0.62-1.59) | | 0.94 (0.51-1.73) | | 1.15 (0.83-1.59) | | 1.15 (0.83-1.60) | |
| **FTC** | 0.50 (0.19-1.34) | | 1.41 (0.85-2.33) | | 1.01 (0.63-1.62) | | 1.06 (0.58-1.95) | | 0.87 (0.63-1.20) | | 0.87 (0.63-1.21) | |
| **TDF** | 0.49 (0.18-1.31) | | 1.29 (0.78-2.13 | | 0.92 (0.58-1.47) | | 1.04 (0.56-1.91) | | 0.88 (0.63-1.22) | | 0.91 (0.66-1.27) | |
| **D4t** | 4.04 (0.20-80.8) | | 1.11 (0.06-21.7) | | 1.10 (0.06-21.5) | | 1.74 (0.09-34.2) | | 0.11 (0.01-2.21) | | 0.19 (0.01-3.72) | |
| NNRTI | | | | | | | | | | | | |
| **EFV** | 0.74 (0.31-1.77) | | 1.63 (0.97-2.74) | | 0.87 (0.54-1.39) | | 0.87 (0.47-1.56) | | 0.99 (0.72-1.37) | | 1.05 (0.76-1.44) | |
| **NVP** | **Without AZT: OR 6.55 (CI 1.14-37.7)**  **With AZT: OR 0.77 (CI 0.25-2.36)*** | | 0.92 (0.50-1.67) | | 1.28 (0.72-2.28) | | 1.52 (0.79-2.92) | | 1.28 (0.88-1.87) | | **1.46 (1.002-2.13)** | |
| PI | | | | | | | | | | | | |
| **DRV** | 1.65 (0.09-29.5) | | 1.56 (0.27-9.19) | | 0.78 (0.13-4.59) | | **4.88 (1.09-21.7)** | | 0.80 (0.21-2.99) | | 0.51 (0.12-2.23) | |
| **LPV/r** | 0.35 (0.06-1.86) | | 0.62 (0.29-1.32) | | 0.76 (0.43-1.36) | | 0.75 (0.32-1.77) | | 0.77 (0.50-1.16) | | 0.65 (0.42-1.01) | |
| **Subtype, OR (95% CI)** | | | | | | | | | | | | |
| **A6** | Reference | | Reference | | Reference | | Reference | | Reference | | Reference | |
| **CRF02_AG** | 0.62 (0.24-1.62) | | 1.19 (0.72-1.99) | | **0.57 (0.36-0.93)** | | **1.91 (1.02-3.55)** | | **31.03 (18.6-51.8)** | | **840.79 (328.5-2152.4)** | |
| **A1+CRF03_AB+G+ CRF 07_BC** | 2.87 (0.48-17.0) | | 1.84 (0.45-7.55) | | 0.35 (0.10-1.25) | | 3.73 (0.88-15.8) | | **8.14 (2.37-27.9)** | | **152.08 (36.13-640.2)** | |

**Supplementary Table 3.**

| **ART components**  (Yes=prescribed) | | **CD4+ Count, median (IQR), cells/mm^3^** | **p-value** | **Viral Load, median (IQR), ×10^3^ copies/mL** | **p-value** |
| --- | --- | --- | --- | --- | --- |
| **ABC** | Administered | 306 (216-405) | 0.69 | 24.7 (6.4-150.3) | 0.87 |
|  | No | 291 (170-451.5) |  | 27.1 (5.2-146) |  |
| **AZT** | Yes | 278 (165-439) | 0.29 | 25.9 (5.3-144) | 0.98 |
|  | No | 301.5 (182.5-451.5) |  | 28.3 (5.3-150.2) |  |
| **3TC** | Yes | 282 (171-423) | 0.22 | 26.4 (5.5-155) | 0.69 |
|  | No | 303 (181-477) |  | 28 (5.2-140) |  |
| **FTC** | Yes | 303 (181-477) | 0.22 | 28 (5.2-140) | 0.69 |
|  | No | 282 (171-423) |  | 26.4 (5.5-155) |  |
| **TDF** | Yes | 302.5 (181-477) | 0.21 | 28.8 (5.3-140.2) | 0.91 |
|  | No | 284.5 (172-421.5) |  | 25.9 (5.4-51.9) |  |
| **D4t** | Yes | 253 (213-314) | 0.68 | 4.1 (0.5-11.8) | 0.08 |
|  | No | 292 (174-448) |  | 27.3 (5.4-150) |  |
| **EFV** | Yes | 300.5 (173.5-460) | 0.42 | 29.2 (5.3-140.1) | 0.88 |
|  | No | 274.5 (180-433) |  | 25.2 (5.3-164) |  |
| **NVP** | Yes | **245 (135-393)** | **0.02** | 26.8 (5-181.5) | 0.63 |
|  | No | **302 (191-456)** |  | 26.7 (5.3-144) |  |
| **DRV** | Yes | 253 (163-294) | 0.30 | 84.5 (2.3-160.2) | 0.92 |
|  | No | 293.5 (176-449) |  | 26.7 (5.4-148) |  |
| **LPV/r** | Yes | **317 (218-466)** | **0.04** | 23.1 (5.3-182.1) | 0.78 |
|  | No | **288.5 (164.5-438)** |  | 28.7 (5.3-149) |  |

**Supplementary Table 4.**

| **Characteristics** | **Overall cohort (n=602)** | **Virologic failure (n=551)** | **Virologic success (n=51)** | **Univariate** | | **Multivariable** | |
| --- | --- | --- | --- | --- | --- | --- | --- |
|  |  |  |  | **OR (95% CI)** | **p-value** | **OR_adj_ (95% CI)** | **p-value** |
| Sex:   - Female (%) - Male (%) | 302 (50.2%)  300 (49.8%) | 280 (50.8%)  271 (49.2%) | 22 (43.1%)  29 (56.9%) | Reference  0.73 (0.42-1.31) | 0.295 | Reference  0.68 (0.36-1.26) | 0.216 |
| Age, years | 39.1±9.1 | 38.9±9.1 | 40.4±9.1 | 0.98 (0.95-1.01) | 0.280 | 0.97 (0.94-1.01) | 0.131 |
| Duration of ART, years | 3.1±2.5 | 3.1±2.5 | 3.6±2.5 | 0.92 (0.83-1.02) | 0.128 | **0.85 (0.76-0.95)** | **0.005** |
| CD4+ cell count, cells/mm^3^ | 332.5±227.5 | 324.5±221.5 | 418.9±272.3 | **0.99 (0.997-0.999)** | **0.005** | **0.99 (0.997-0.999)** | **0.002** |
| ART regimen^a^ (%):   - ABC - AZT - 3TC - FTC - TDF - D4t - EFV - NVP - DRV - LPV/r | 86 (14.3%)  286 (47.5%)  355 (59.0%)  247 (41.0%)  250 (41.5%)  3 (0.5%)  316 (52.5%)  143 (23.8%)  8 (1.3%)  106 (17.6%) | 81 (14.7%)  267 (48.5%)  331 (60.1%)  220 (39.9%)  223 (40.5%)  2 (0.4%)  288 (52.3%)  133 (24.1%)  6 (1.1%)  97 (17.6%) | 5 (9.8%)  19 (37.3%)  24 (47.1%)  27 (52.9%)  27 (52.9%)  1 (2.0%)  28 (54.9%)  10 (19.6%)  2 (3.9%)  9 (17.7%) | 1.46 (0.59-3.65)  1.57 (0.87-2.81)  1.69 (0.95-2.99)  0.59 (0.33-1.05)  0.61 (0.34-1.07)  0.15 (0.02-1.18)  0.90 (0.51-1.59)  1.26 (0.62-2.55)  0.24 (0.04-1.04)  0.96 (0.46-2.01) | 0.414  0.133  0.072  0.072  0.085  0.072  0.726  0.519  0.057  0.913 | **2.50 (1.32-4.71)** | **0.005** |
| Mutations (%):   - I13V - N37D - I93L - V77I - Q174K | 406 (67.4%)  225 (37.4%)  197 (32.7%)  93 (15.5%)  521 (86.5%) | 381 (69.2%)  213 (38.7%)  172 (31.2%)  80 (14.5%)  484 (87.8%) | 25 (49.0%)  12 (23.5%)  25 (49.0%)  13 (25.5%)  37 (72.6%) | **2.33 (1.31-4.15)**  **2.05 (1.05-3.99)**  **0.47 (0.26-0.84)**  **0.49 (0.25-0.97)**  **2.73 (1.40-5.32)** | **0.004**  **0.036**  **0.011**  **0.041**  **0.003** | **2.56 (1.41-4.66)**  **0.42 (0.21-0.86)**  **3.14 (1.58-6.25)** | **0.002**  **0.017**  **0.001** |

**TABLE LEGENDS**

**Supplementary Table 1.** The most frequently occurring ARTRM among the study participants.

**Supplementary Table 2.** The association between CD4 count, Viral load, ART components, Subtype and single mutations. (+) presence of the mutation; (-) absence of the mutation ^a^ comparison of PLWH administered with a specific ART component to the PLWH, who are not administered with this medication OR odds ratio; CI 95% confidence interval. * interaction between Zidovudine (AZT) and Nevirapine (NVP)

**Supplementary Table 3.** The association between ART component and CD4 count or Viral load

**Supplementary Table 4.** The association between characteristics of 602 PLWH in the study and virologic failure (>1000 copies/ml). OR odds ratio; CI 95% confidence interval; ORadj adjusted odds ratio ^a^ comparison of PLWH administered with a specific ART component to the PLWH, who are not administered with this medication
